# Overexpression of caudal type homeobox transcription factor 2 inhibits the growth of the MGC-803 human gastric cancer cell line *in vivo*

**DOI:** 10.3892/mmr.2015.3413

**Published:** 2015-03-04

**Authors:** WEIYUAN WEI, LEI LI, XIAOTONG WANG, LINHAI YAN, WENLONG CAO, ZEXU ZHAN, XIAOSHI ZHANG, HAN YU, YUBO XIE, QIANG XIAO

**Affiliations:** 1 Department of Surgery, The First Affiliated Hospital of Guangxi Medical University, Nanning, Guangxi 530021, P.R. China; 2 Department of Surgery, People’s Hospital of Guangxi Zhuang Autonomous Region, Nanning, Guangxi 530021, P.R. China; 3 Department of Anesthesiology, The First Affiliated Hospital of Guangxi Medical University, Nanning, Guangxi 530021, P.R. China

**Keywords:** caudal type homeobox transcription factor 2, gastric cancer, nude mice model, nuclear factor-κB pathway

## Abstract

Caudal type homeobox transcription factor 2 (CDX2) is important in intestinal cell fate specification and multiple lines of evidence have substantiated that CDX2 is important in carcinogenesis of the digestive tract. The CDX2 regulatory network is intricate and remains to be fully elucidated in gastric cancer. The aim of the present study was to examine the effects of CDX2 on the growth of the MGC-803 human gastric cancer cell line *in vivo*, and to elucidate the mechanism involved. The effects of the overexpression of CDX2 in xenograft tumors of MGC-803 cells was investigated in nude mice through the injection of CDX2 recombinant lentiviral vectors. The tumor size was measured using vernier callipers. The expression levels of CDX2, survivin, B-cell lymphoma 2 (Bcl-2), Bcl-2-associated X protein (Bax), cyclin D1, s-phase kinase-associated protein 2 (Skp2) and c-Myc in the tumor cells were analyzed by western blotting and semi-quantitative reverse transcription polymerase chain reaction. The apoptotic rates were determined using a terminal deoxynucleotidyl transferase-mediated dUTP-biotin nick end labeling assay. The overexpression of CDX2 was observed in the group subjected to the injection of CDX2 recombinant lentiviral vectors. CDX2 had an inhibitory effect on the MGC-803 human gastric cancer cell line and promoted tumor cell apoptosis *in vivo*. Furthermore, the overexpression of CDX2 upregulated the expression of Bax and downregulated the expression levels of survivin, Bcl-2, cyclin D1, Skp2 and c-Myc in the tumor tissues. These results indicated that CDX2 may serve as a tumor suppressor in gastric cancer, and inhibits gastric cancer cell growth by suppressing the nuclear factor-κB signaling pathway.

## Introduction

Gastric cancer is among the most common type of cancer worldwide and is currently the third most common type of cancer, although the incidence is decreasing (1). Despite novel treatment strategies, including perioperative chemotherapy and adjuvant chemoradiation using external radiotherapy, gastric cancer is usually diagnosed at an advanced stage and the prognosis remains poor (2). Thus, an improved understanding of the molecular events involved in the development and progression of gastric cancer may lead to novel treatment methods with improved efficacy.

Caudal type homeobox transcription factor 2 (CDX2) is a member of the Cdx gene family and is an intestine-specific homeobox transcription factor, which is highly expressed in the intestinal epithelium of adult animals, where it is responsible for directing the differentiation of intestinal epithelial cells (3,4). Importantly, CDX2 is associated with the development of intestinal metaplasia of the stomach and with gastric carcinogenesis (5,6). Certain studies have demonstrated that there are significant correlations between CDX2 and intestinal-type adenocarcinoma (7,8). In addition, a previous biological study demonstrated that CDX2 may be important in gastric tumorigenesis (9), whereas another study suggested that CDX2 is a tumor suppressor (10).

To the best of our knowledge, no comprehensive studies have been performed to assess the overexpression of CDX2 in gastric cancer. In our previous study, the overexpression of CDX2 exhibited a significant effect on cell growth and proliferation in an *in vitro* cell model of gastric cancer (11). However, the molecular mechanisms underlying the overexpression of CDX2, which inhibit cell growth and increase the levels of apoptosis remain to be fully elucidated. The aim of the present study was to evaluate the effects of the overepxression of CDX2 on the growth and level of apoptosis in MGC-803 cells *in vivo* by administering nude mice with intratumoral injections of a recombinant CDX2 lentivirus. In addition, to reveal the possible underlying mechanisms, the effects of the overexpression of CDX2 on the mRNA and protein expression levels of B-cell lymphoma 2 (Bcl-2), Bcl-2-associated X protein (Bax), survivin, cyclin D1, S-phase kinase-associated protein 2 (Skp2) and c-Myc were examined in MGC-803 cells *in vivo*.

## Materials and methods

### Antibodies

Specific rabbit anti-human polyclonal antibodies to CDX2 (#12306), c-Myc (#5605), Skp2 (#2652), Bax (#5023), Bcl-2 (#2827), cyclin D1 (#2978), survivin (#2808) and GAPDH (#2118) were provided by Cell Signaling Technology, Inc. *(Beverly, MA, USA)*. Infrared-labeled secondary goat anti-rabbit antibodies to IRDye 800 were obtained from Li-Cor Biosciences (Lincoln, NE, USA). All the above antibodies were used by diluted 1000 times in western blot analysis.

### Cell culture

The MGC-803 human gastric carcinoma cell line and 293T human embryonic kidney cells were provided by the Cell Bank of Shanghai Institute of Cell Biology, Chinese Academy of Sciences (Shanghai, China). The cells were maintained at 37°C in an atmosphere containing 5% CO_2_ in Dulbecco’s modified Eagle’s Medium, supplemented with 10% fetal bovine serum, 100U/ml penicillin and 100 *μ*g/ml streptomycin.

### Construction of the CDX2 recombinant lentiviral vectors

The lentivitus overexpressing the CDX2 gene was constructed at Shanghai Genechem Co., Ltd. (Shanghai, China). The lentiviral vector system consisted of a GV208, pHelper 1.0 vector and pHelper 2.0 vector prior to packaging and was provided by Shanghai Genechem Co., Ltd. (Shanghai, China). The full length of the human CDX2 gene (NCBI ID, NM_001265.4), which was indicated by enhanced green fluorescent protein (GFP), which was encoded into the GV208 vector. The three vectors were cotransfected into 293T cells (3×10^4^/ml) in serum-free medium using Lipofectamine 2000 (Invitrogen Life Technologies, Carlsbad, CA, USA). The medium was replaced with complete medium following 8 h incubation at 37°C. The high-titer recombinant lentiviral vectors carrying CDX2 were harvested 48 h following transfection.

### Xenograft tumor model

The animals used in the present study were BALB/c nude male mice (4 weeks old), which were purchased from Guangxi Animal Center (Nanning, China). The number of nude mice was six in each group, weighing between 20 and 24 g, and they were fed under specific pathogen-free conditions. All procedures were in accordance with the National Institutes of Health Guide for the Care and Use of Laboratory Animals (National Institutes of Health, Bethesda, MD, USA). Tumors were established in the mice via a single subcutaneous injection of 4×10^7^ MGC-803 cells into the armpit region. The study was approved by the ethics committee of the First Affiliated Hospital of Guangxi Medical University, (Guangxi, China).

### Treatment of the MGC-803 tumor in nude mice

When the tumors had reached a diameter of ~5 mm, the mice were randomized into three groups: Lentivirus (LV)-GFP-CDX2, LV-GFP-negative control (NC) and phosphate-buffered saline (PBS; Beyotime Institute of Biotechnology, Shanghai, China). Each group contained eight mice (n=8). The animals were administered with an intratumoral injection of either the LV-GFP-CDX2 or LV-GFP-NC at a titer of 10^8^ transducing units in 100 *μ*l PBS, while the control group of mice received an equal volume of PBS. Subsequent to the first injection, the animals were administered with a similar injection every 2 days. The mouse body weight, the quantity of water and food intake, vital signs and living status were assessed daily. The tumor volume was measured and calculated as follows: The longer diameter, ‘a’, and the shortest diameter, ‘b’, of the tumors were measured using digital calipers, and the tumor volume (TV) was calculated using the following equation: TV = a × b^2^/2. The relative tumor volume (RTV) was calculated using the formula: RTV = V_t_ / V_0_, in which V_0_ is the TV on the day when the treatment was administered, and V_t_ is the TV of the subsequent measurement. Following the tumor cell injections (15 days), the animals were sacrificed by cervical dislocation and the tumors were then analyzed.

### Reverse transcription semi-quantitative-polymerase chain reaction (RT-sqPCR)

The total RNA was extracted from the tumor tissues using TRIzol reagent (Sigma-Aldrich, St. Louis, MO, USA), according to the manufacturer’s instructions. cDNA was generated from a DNase-1-treated RNA template with 0.2 *μ*g random hexamer primers (Takara Bio, Inc., Tokyo, Japan) and 200 units RevertAid H-Minus M-MuLV reverse transcriptase enzyme (Roche, Basel, Switzerland). The primer sequences used to specifically amplify the genes of interest are shown in Table I. The cDNA (2 *μ*l) produced was added to 10 *μ*l Taq Premix and the upstream and downstream primers (1 *μ*l each). RT-qPCR was performed as follows: 1 cycle at 94°C for 5 min, 30 cycles at 94°C for 30 sec for denaturation, 56°C for 30 sec for annealing, 68°C for 45 sec for extension and 1 cycle 5 min at 72°C, according to the RT-qPCR amplification kit (Takara Bio, Inc.) manufacturer’s instructions. The amplified PCR products were run on 1.5% agarose gels and visualized under UV light following ethidium bromide (0.5 *μ*g/ml; Beyotime Institute of Biotechnology) staining at room temperature (25°C) for 20 min.

### Western blot analysis

The tumor tissues were homogenized for tissue lysate extraction, the tissue lysates were centrifuged and the supernatants were collected. Equal quantities (150 *μ*g) of protein were heated to 100°C for 5 min with Laemmli sample buffer (Beyotime Institute of Biotechnology), then separated on 12% SDS-PAGE gels (Beyotime Institute of Biotechnology) and transferred onto polyvinylidene difluoride membranes. The entire process was performed using Bio-Rad equipment (Bio-Rad Laboratories, Inc., Hercules, CA, USA) according to the manufacturer’s instructions. The membrane was probed with the primary antibody (1:1,000) and incubated overnight at 4°C. The blots were washed three times in PBS with Tween 20 prior to incubation with species-appropriate, peroxidase-conjugated secondary antibodies for 1 h. The blots were then washed again three times in PBS with Tween 20. The net intensities of the bands were quantified using Odyssey software version 3.0 (Li-Cor Biosciences, Lincoln, NE, USA).

### In situ analysis of MGC-803 tumor cell apoptosis using a terminal deoxynucleotidyl transferase-mediated dUTP-biotin nick end labeling (TUNEL) assay

Tissue samples were fixed in 4% buffered paraformaldehyde at 4℃ for 48 h and then processed for paraffin embedding. The procedures of paraffin embedding were dehydration and waxdip. Paraffin-embedded (Beyotime Institute of Biotechnology) sections were prepared for hematoxylin and eosin (Beyotime Institute of Biotechnology) staining. The levels of tumor tissue necrosis were determined by comparing the surface of necrotic areas with that of the whole tumor. Levels of apoptosis were determined using a TUNEL assay kit, according to the manufacturer’s instructions. Briefly, the cells were rinsed with PBS twice for 3 min, prior to the addition of 50 *μ*l TUNEL cocktail on test sections. Labeling solution (40 *μ*l) was added to control sections on one slide and PBS was added to the control sections on other slides, and incubated in a humidified chamber for 60 min at 37ºC in the dark. A sample was considered positive when it contained 25 positively stained cells in every 100 tumor cells, which was calculated from five randomly selected fields for each specimen. The stained tissue sections were visualized by microscopy (CP-111-2; magnification, x400; Jenco International, Protland, OR, USA).

### Statistical analysis

Data are expressed as the mean ± standard error of the mean and were analyzed using SPSS version 13.0 (SPSS, Inc., Chicago, IL, USA). One-way analysis of variance was used to measure statistical significance among groups, followed by the Student-Newman-Keuls test. P<0.05 was considered to indicate a statistically significant difference.

## Results

### Construction and identification of pGCL-GFP-CDX2 lentiviral vectors

The positive clones were confirmed by DNA sequence analysis (data not shown) and it was demonstrated that the RNA coding frames and frame sequences were correct and that the recombinant pGCL-GFP-CDX2 and pGCL-GFP-NC plasmids had been constructed successfully.

### Determination of lentiviral titers

**Figure 1:**
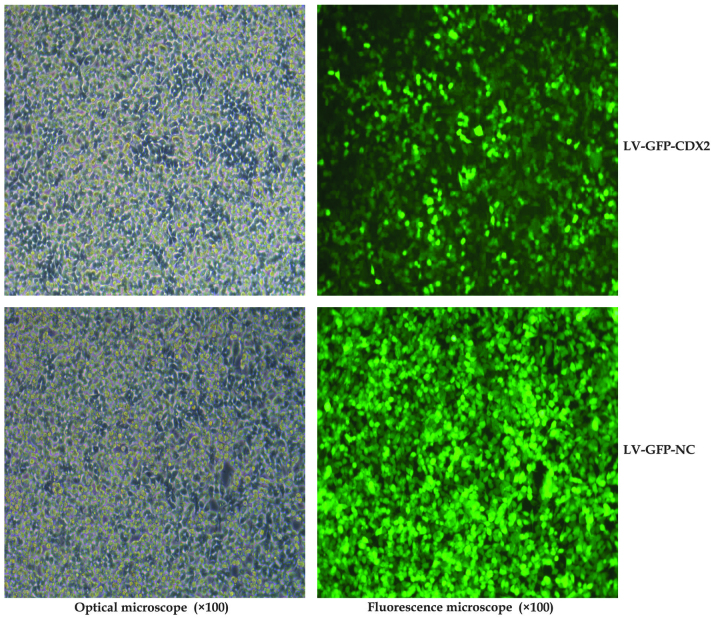
Determination of lentivirus titers. The viral titers of recombinant lentivirus transfected into 293T cells were determined by end point dilution, through counting the numbers of fluorescent green-labeled cells under a fluorescence microscope (magnification, x100). The viral dilution factor was 1:1,000. LV-GFP, lentivirus-green fluorescent protein; NC, negative control; CDX2, caudal type homeobox transcription factor 2.

A lentivirus targeting CDX2 and an NC vector (LV-GFP-CDX2, and LV-GFP-NC, respectively) were produced by co-transfection with a packaging vector (pHelper1.0) and a vesicular stomatitis virus glycoprotein expression plasmid (pHelper2.0) into the 293T cells. As shown in Fig. 1, the GFP-labeling results indicated that the lentiviral vectors were suitably transfected for use in the present study.

### Overexpression of CDX2 inhibits MGC-803 tumor growth

**Figure 2:**
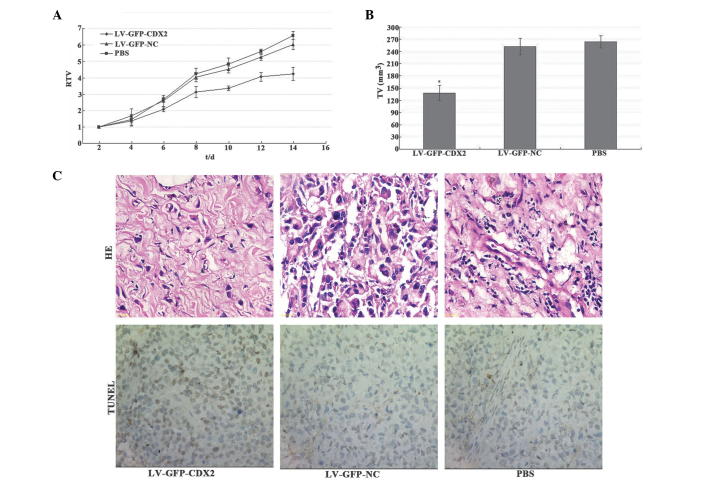
Overexpression of CDX2 inhibits tumor growth and induces tumor cell apoptosis in LV-GFP-CDX2-treated mice. (A) Relative tumor volume growth curve revealed significant growth tendencies in the PBS and LV-GFP-NC groups, while the MGC-803 tumor growth in the LV-GFP-CDX2 group was markedly inhibited. (B) Tumor volumes in the LV-GFP-CDX2 groupe were smaller compared with those in the control group 14 days after tumor injection (^*^P<0.05). (C) Tumor cell apoptosis was assessed using a TUNEL assay and HE staining, revealing that the MGC-803 tumor cells in the LV-GFP-CDX2 group had higher levels of apoptosis compared with the LV-GFP-NC and PBS groups (magnification, x400). CDX2, vaudal type homeobox transcription factor 2; LV-GFP, lentivirus-green fluoresent protein; PBS, phosphate-buffered saline; NC, negative control; TV, tumor volume; RTV, relative TV; TUNEL, terminal deoxynucleotidyl transferase-mediated dUTP-biotin nick end labeling; HE, hematoxylin and eosin.

As shown in Fig. 2A, the tumor growth curves indicated that mice treated with LV-GFP-CDX2 exhibited significant inhibition of tumor growth when compared with those treated with the LV-GFP-NC control vector or PBS (P<0.05). The tumor volumes in the mice in the LV-GFP-CDX2 group were significantly smaller compared with those of the control groups (P<0.05) at 15 days post-tumor injection, whereas no difference was identified between the Lv-GFP-NC and PBS groups (P>0.05; Fig. 2B). These results indicated that overexpression of CDX2 effectively inhibited MGC-803 tumor growth *in vivo*.

### Overexpression of CDX2 induces MGC-803 tumor cell apoptosis

As shown in Fig. 2C, the percentage of apoptotic tumor cells in the LV-CDX2-GFP group was 17.32±2.5%, which was significantly higher compared with that observed in the LV-GFP-NC (7.2±1.7%) and PBS (6.6±1.8%) groups, demonstrated using the TUNEL method (P<0.05). These results suggested that overexpression of CDX2 effectively promoted MGC-803 tumor cell apoptosis *in vivo*.

### mRNA and protein expression levels of CDX2 are increased in MGC-803 tumor tissues

**Figure 3:**
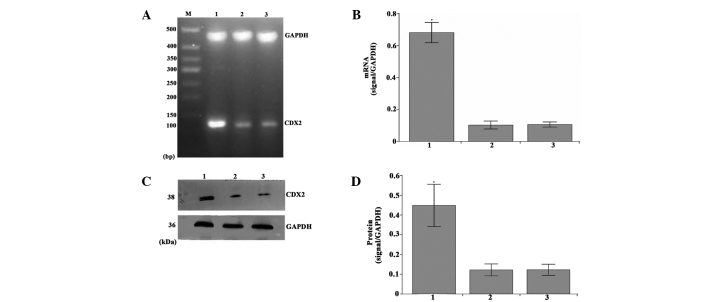
Overexpression of CDX2 mRNA and protein in the LV-GFP-CDX2 group. (A) Reverse transcription semi-quantitative polymerase chain reaction analysis of CDX2 and GAPDH in the MGC-803 tumor tissues from the LV-GFP-CDX2, LV-GFP-NC and PBS groups. M, 500 bp marker. (B) mRNA expression levels of CDX2 were measured in the three groups, normalized to GAPDH and presented as the mean ± standard error of the mean (n=8 in each group). (C) Western blot analysis of the protein expression levels of CDX2 and GAPDH in the MGC-803 tumor tissues from the three groups. (D) Protein expression levels of CDX2 were measured in the three groups, normalized to GAPDH and presented as the mean ± standard error of the mean (n=8 in each group). Lanes: 1, LV-GFP-CDX2 group; 2, LV-GFP-NC group; 3, PBS group, GAPDH: internal control mRNA and protein.^*^P<0.05 compared with LV-GFP-NC and PBS group, using analysis of variance and Student-Newman-Keuls analyses. CDX2, caudal type homeobox transcription factor 2; LV-GFP, lentivirus-green fluoresent protein; PBS, phosphate-buffered saline; NC, negative control.

Densitometric analysis revealed that mRNA and protein expression levels of CDX2 in the LV-GFP-CDX2 group were higher compared with those of the two control groups (P<0.05; Fig. 3A–D). These results suggested that the nude mouse model overexpressing CDX2 had been constructed successfully by injection with the CDX2 recombinant lentiviral vectors.

### Overexpression of CDX2 decreases the expression levels of c-Myc, Skp2, Bcl-2, cyclin D1 and survivin, and increases the expression of Bax

**Figure 4:**
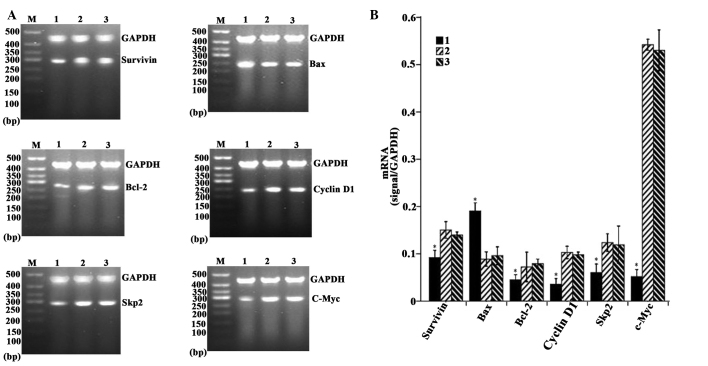
Overexpression of CDX2 induces downregulation in the mRNA expression levels of c-Myc, Skp2, Bcl-2, cyclin D1 and survivin and upregulation in the expression of Bax. (A) Reverse transcription semi-quantitative polymerase chain reaction analysis of c-Myc, Skp2, Bcl-2, cyclin D1, survivin, Bax and GAPDH in the MGC-803 tumor tissues from the LV-GFP-CDX2, 2, LV-GFP-NC and PBS groups. M, 500 bp marker (B) mRNA expression levels of c-Myc, Skp2, Bcl-2, cyclinD1, survivin and Bax were measured in the three groups, normalized to those of GAPDH and presented as the mean ± standard error of the mean (n=8 in each group). 1, LV-GFP-CDX2 group; 2, LV-GFP-NC group; 3, PBS group; GAPDH: internal control. ^*^P<0.05, compared with the LV-GFP-NC and PBS groups, using analysis of variance and Student-Newman-Keuls analyses. Bcl-2, B-cell lymphoma 2; Bax, Bcl-2-associated X protein; Skp2, S-phase kinase-associated protein 2; CDX2, caudal type homeobox transcription factor 2; LV-GFP, lentivirus-green fluoresent protein; PBS, phosphate-buffered saline.

**Figure 5:**
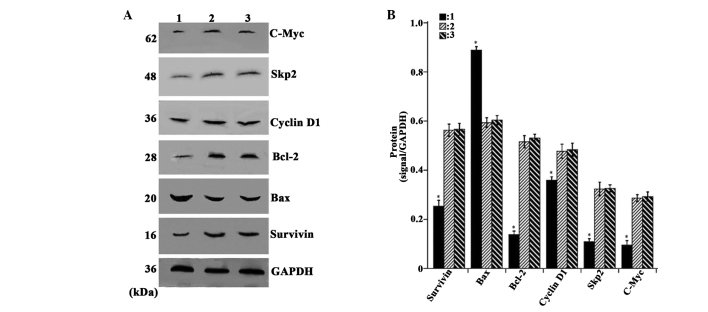
Overexpression of CDX2 induces downregulation of the protein expression levels of c-Myc, Skp2, Bcl-2, cyclinD1 and survivin and upregulation of Bax. (A) Western blot analysis of c-Myc, Skp2, Bcl-2, cyclin D1, survivin, Bax and GAPDH in the MGC-803 tumor tissue from the LV-GFP-CDX2, LV-GFP-NC and PBS groups. (B) Protein expression levels of c-Myc, Skp2, Bcl-2, cyclin D1, survivin and Bax were measured in the three groups, normalized to those of GAPDH and expressed as the mean ± standard error of the mean (n=8 in each group). 1, LV-GFP-CDX2 group; 2, LV-GFP-NC group; 3, PBS group; GAPDH: internal control. ^*^P<0.05, compared with the LV-GFP-NC and PBS group, using analysis of variance and Student-Newman-Keuls analyses. Bcl-2, B-cell lymphoma 2; Bax, Bcl-2-associated X protein; Skp2, S-phase kinase-associated protein 2; CDX2, caudal type homeobox transcription factor 2; LV-GFP, lentivirus-green fluoresenct protein; PBS, phosphate-buffered saline.

As shown in Fig. 4A and B, the densitometric analysis revealed that the mRNA expression levels of c-Myc, Skp2, Bcl-2, cyclin D1 and survivin in the LV-GFP-CDX2 group were lower, while the expression of Bax was higher compared with the LV-GFP-NC and PBS groups (P<0.05). In addition, as shown in Fig. 5A and B, the densitometry revealed that the protein expression levels of c-Myc, Skp2, Bcl-2, cyclin D1 and survivin in the LV-GFP-CDX2 group was lower, that of while Bax was higher compared with the LV-GFP-and PBS groups (P<0.05). These results suggested that the overexpression of CDX2 effectively decreased the expression levels of c-Myc, Skp2, Bcl-2, cyclin D1, survivin, and increased the expression of Bax in the MGC-803 tumor cells *in vivo*.

## Discussion

CDX2 is a nuclear transcription factor, which is important in embryologic development and in the differentiation of the intestinal tract epithelium (12). It is also highly expressed in epithelial tumors of the gastrointestinal tract (13) and, for this reason, its role in tumorigenesis has become an important area of investigation. Although several lines of evidence have indicated that CDX2 is a potential tumor suppressor gene in ovarian, gallbladder, colon and gastric cancer (12,14–17), the mechanisms associating the overexpression of CDX2 with gastric cancer remain to be elucidated.

In the present study, a marked antitumoral effect of the overexpression of CDX2 on MGC-803 cells was observed *in vivo*. Tumor growth was suppressed and tumor apoptosis was increased in nude mice when the CDX2 mRNA and protein were overexpressed via lentiviral vector-mediated overexpression of CDX2. These findings were concordant with our previous study, which observed that the overexpression of CDX2 inhibits the progression of gastric cancer *in vitro* (11,18). Therefore, lentiviral vector-mediated overexpression of CDX2 may be used as a potent and specific therapeutic tool for the treatment of gastric cancer. In addition, the present study revealed that overexpression of CDX2 decreased the expression levels of survivin, Bcl-2, cyclin D1, Skp2 and c-Myc, and increased the expression of Bax.

Previous studies have confirmed that the Bax, Bcl-2, cyclin D1, c-Myc, Skp2 and survivin genes are associated with cell proliferation, cell apoptosis and tumor development (19–22). Takahashi *et al* (23) suggested that CDX2 inhibited the gene expression of exogenous nuclear factor (NF)-κB-induced luciferase in a dose-dependent manner. Furthermore, Yang *et al* (24) and Saha *et al* (25) demonstrated that NF-κB induces the expression of genes involved in cell proliferation (cyclin D1 and c-Myc) and anti-apoptotic (survivin and Bcl-2), while it inhibits the expression of the pro-apoptotic gene, Bax. In addition, the NF-κB signaling pathway regulates the cell cycle by binding of the NF-κB subunits to the cyclin D1, c-Myc and Skp2 promoters, which are concomitant with a switch from coactivator to corepressor recruitment (26). This suggests that the overexpression of CDX2 may directly or indirectly modulate the transcriptional activity of downstream genes (Bax, Bcl-2, cyclin D1, Skp2, c-Myc and survivin) by inhibiting the gene expression of NF-κB.

Downregulation of the NF-κB signaling pathway induces downregulation of the anti-apoptotic gene, Bcl-2 and upregulation of the pro-apoptotic gene, Bax (27). The Bcl-2 family of proteins represent essential targets in cancer therapy (28). The Bax, Bcl-2 homologous antagonist killer and Bcl-2 related ovarian killer pro-apoptotic and Bcl-2, Bcl-extra large and myeloid cell leukemia 1 anti-apoptotic members of the Bcl-2 family may promote or inhibit apoptosis through the formation of heterodimers among these proteins (29). Therefore, the ratio between the pro-apoptotic Bax and anti-apoptotic Bcl-2 proteins is an important determinant of cell survival and death. In the present study, upregulation of the pro-apoptotic protein, Bax and downregulation of the anti-apoptotic protein, Bcl-2 were observed in the LV-CDX2-GFP group, and gray scale value analysis revealed a significantly higher Bax/Bcl-2 ratio in the treatment group compared with the untreated controls. A higher Bax/Bcl-2 ratio has been reported to be a cause of cell death (30). This suggests that the overexpression of CDX2 induced apoptosis by altering the Bax/Bcl-2 ratio to suppress gastric cancer growth.

Barré *et al* (26) observed that NF-κB subunits regulate the gene expression of the cyclin D1, c-Myc and Skp2, and downregulation of NF-κB can result in a change in the function of the NF-κB-binding site, resulting in repression of the cyclin D1, c-Myc and Skp2 gene promoter. Cyclin D1, c-Myc and Skp2 are cell cycle regulators, and the cell cycle is arrested through suppression of the expression of cyclin D1, c-Myc and Skp2 (31-33). Therefore, the overexpression of CDX2 may also suppress the cell cycle through the indirect suppression of the expression of cyclin D1, c-Myc and Skp2 through the NF-κB signaling pathway. In addition, cell immortalization is a basic step in tumor growth (34). Therefore, control of the cell cycle may be an important mechanism in the suppression of tumor growth by CDX2 in gastric cancer.

A previous study demonstrated that survivin is down-regulated via the NF-κB-mediated signaling pathway, thus inhibiting the growth of cancer cells (35). Survivin is an important factor in cell division, and the separation of chromatin in mitosis may be faulty in cancer cells lacking expression of the survivin gene (36,37). The cell cycle checkpoint mechanism activates following mitotic dysfunction, which promotes apoptosis in abnormal cells (38). The present study demonstrated that the overexpression of CDX2 significantly inhibited the growth of transplanted tumors and promoted cell apoptosis, which may be attributed to the indirect downregulation of survivin by CDX2, by inhibiting the gene expression of NF-κB.

In conclusion, the CDX2/NF-κB signaling pathway is an unusually structured network, by which CDX2 inhibits the growth of MGC-803 cells *in vivo*. This may explain an important aspect of the mechanism by which the overexpression of CDX2 contributes to the suppression of gastric cancer cell growth.

## Figures and Tables

**Table I:** Sequences of the primers used for reverse transcription semi-quantitative polymerase chain reaction.

Gene	Primer	Sequence	PCR product (bp)
CDX2	Forward	5′- CGGCAGCCAAGTGAAAAC-3′	217
	Reverse	5′-GATGGTGATGTAGCGACTGTAGTG-3′	
Survivin	Forward	5′-AAATGCACTCCAGCCTCTGT-3′	311
	Reverse	5′-TGTCGAGGAAGCTTTCAGGT-3′	
Bax	Forward	5′-CCAAGAAGCTGAGCGAGTGT-3′	269
	Reverse	5′-CCGGAGGAAGTCCAATGTC-3′	
Bcl-2	Forward	5′-GACTTCGCCGAGATGTCCAG-3′	259
	Reverse	5′-CATCCCAGCCTCCGTTATCC-3′	
Cyclin D1	Forward	5′-CCCTCGGTGTCCTACTTCAA-3′	237
	Reverse	5′-GGGGATGGTCTCCTTCATCT-3′	
Skp2	Forward	5′-GCTGCTAAAGGTCTCTGGTGT-3′	291
	Reverse	5′-AGGCTTAGATTCTGCAACTTG-3′	
C-Myc	Forward	5′-TTCTCTCCGTCCTCGGATTC-3′	282
	Reverse	5′-GTAGTTGTGCTGATGTGTGG-3′	
GAPDH	Forward	5′-ACCACAGTCCATGCCATCAC-3′	450
	Reverse	5′-TCACCACCCTGTTGCTGTA-3′	

Bcl-2, B-cell lymphoma 2; CDX2, caudal type homeobox transcription factor 2; Bax, Bcl-2-associated X protein; Skp2, S-phase kinase-associated protein 2.
